# A population-wide analysis of the familial risk of suicide in Utah, USA

**DOI:** 10.1017/S0033291721003020

**Published:** 2023-03

**Authors:** Amanda V. Bakian, Danli Chen, Chong Zhang, Heidi A. Hanson, Anna R. Docherty, Brooks Keeshin, Douglas Gray, Ken R. Smith, James A. VanDerslice, David Z. Yu, Yue Zhang, Hilary Coon

**Affiliations:** 1 Department of Psychiatry, Huntsman Mental Health Institute, University of Utah School of Medicine, Salt Lake City, UT, USA; 2 Department of Family and Preventive Medicine, University of Utah School of Medicine, Salt Lake City, UT, USA; 3 Study Design & Biostatics Center, Utah Clinical & Translational Science Institute, Salt Lake City, Utah, USA; 4 Department of Surgery, University of Utah School of Medicine, Salt Lake City, UT, USA; 5 Population Sciences, Huntsman Cancer Institute, Salt Lake City, UT, USA; 6 Scientific Computing Institute, University of Utah, Salt Lake City, UT, USA; 7 Department of Pediatrics, University of Utah School of Medicine, Salt Lake City, UT, USA; 8 Department of Family and Consumer Studies, University of Utah, Salt Lake City, UT, USA; 9 Department of Internal Medicine, University of Utah School of Medicine, Salt Lake City, UT, USA

**Keywords:** suicide, familial risk, Utah, unified model, Cox regression, population attributable fraction

## Abstract

**Background:**

The degree to which suicide risk aggregates in US families is unknown. The authors aimed to determine the familial risk of suicide in Utah, and tested whether familial risk varies based on the characteristics of the suicides and their relatives.

**Methods:**

A population-based sample of 12 160 suicides from 1904 to 2014 were identified from the Utah Population Database and matched 1:5 to controls based on sex and age using at-risk sampling. All first through third- and fifth-degree relatives of suicide probands and controls were identified (*N* = 13 480 122). The familial risk of suicide was estimated based on hazard ratios (HR) from an unsupervised Cox regression model in a unified framework. Moderation by sex of the proband or relative and age of the proband at time of suicide (<25 *v.* ⩾25 years) was examined.

**Results:**

Significantly elevated HRs were observed in first- (HR 3.45; 95% CI 3.12–3.82) through fifth-degree relatives (HR 1.07; 95% CI 1.02–1.12) of suicide probands. Among first-degree relatives of female suicide probands, the HR of suicide was 6.99 (95% CI 3.99–12.25) in mothers, 6.39 in sisters (95% CI 3.78–10.82), and 5.65 (95% CI 3.38–9.44) in daughters. The HR in first-degree relatives of suicide probands under 25 years at death was 4.29 (95% CI 3.49–5.26).

**Conclusions:**

Elevated familial suicide risk in relatives of female and younger suicide probands suggests that there are unique risk groups to which prevention efforts should be directed – namely suicidal young adults and women with a strong family history of suicide.

## Introduction

Suicide is the 10th leading cause of death in the USA (Hedegaard, Curtin, & Warner, 2018). While the risk of other top causes of mortality has declined recently in the USA, suicide rates increased by 30% between 1999 and 2016 (Stone et al., 2018). Suicide's etiology is complex with predisposing, mediating, and short-term risk factors from genetic and environmental sources implicated in its causal pathway (Turecki, 2014). Low predictive performance of existing prediction tools (Belsher, Smolenski, & Pruitt, 2019) and a lack of effective evidence-based interventions for suicide mortality (Nelson et al., 2017; Riblet, Shiner, Young-Xu, & Watts, 2017) indicate the ongoing need for improved understanding of suicide's risk factors.

The aggregation of suicide mortality (herein referred to as ‘suicide’) in families is a strong risk factor for suicide with evidence originating largely from twin (Juel-Nielsen & Videbech, 1970; Pedersen & Fiske, 2010; Roy, Segal, Centerwall, & Robinette, 1991), adoption (Kendler, Ohlsson, Sundguist, Sundguist, & Edwards, 2020; Petersen, Sørensen, Andersen, Mortensen, & Hawton, 2013; Schulsinger, Kety, Rosenthal, & Wender, 1979; von Borczyskowski, Lindblad, Vinnerljung, Reintjes, & Hjern, 2011), and population-based family studies (Agerbo, 2005; Agerbo, Nordentoft, & Mortensen, 2002; Cheng et al., 2014; Egeland & Sussex, 1985; Garssen, Deerenberg, Mackenbach, Kerkhof, & Kunst, 2011; Kim et al., 2005; Qin & Mortensen, 2003; Qin, Agerbo, & Mortensen, 2002, 2003; Runeson & Åsberg, 2003; Tidemalm, Runeson, & Waern, 2011). While twin and adoption studies offer robust designs for the investigation of familial suicide risk among close relatives, population-based family studies often include large sample sizes, which allow for the calculation of familial suicide risks in specific kinships across a broad range of relatives. The preferred family study is prospective and uses random sampling thereby minimizing the risk of selection bias (Hopper, Bishop, & Easton, 2005). To date, research into the familial risk of suicide using population-based family study designs has largely been limited to work done in Northwestern European countries using data linked across health and multi-generational registries (but see Cheng et al., 2014; Kim et al., 2005). These studies have primarily measured the familial aggregation of suicide in first-degree relatives (Agerbo, 2005; Agerbo et al., 2002; Garssen et al., 2011; Qin & Mortensen, 2003; Qin et al., 2002, 2003; Runeson & Åsberg, 2003) with a single study (Tidemalm et al., 2011) examining familial risk in second- and third-degree relatives (e.g. cousins). In combination, population-based family studies report a two (Agerbo, 2005; Agerbo et al., 2002; Garssen et al., 2011; Qin & Mortensen, 2003; Qin et al., 2002, 2003; Runeson & Åsberg, 2003; Tidemalm et al., 2011) to 15-fold [in monozygotic twins (Tidemalm et al., 2011)] increase in the risk of suicide among first-degree relatives of suicide probands. Some of this work suggests that suicide's familial liability may be higher in female relatives compared to males (Cheng et al., 2014; Qin et al., 2003; Qin & Mortensen, 2003) especially if the suicide proband is also female or young at time-of-death (Cheng et al., 2014; Garssen et al., 2011; Qin & Mortensen, 2003). Little has been reported on the familial liability of suicide across specific kinships in more distantly related relatives.

In the USA, data resources comparable to the health registries in Northwestern Europe are not widely available for examining the patterns of familial suicide transmission using a population-based family study design. The exception being the pioneering work done in the Old Order Amish in which all suicides in a 100-year period were identified (*n* = 26 deaths) and linked to their extended pedigrees (Egeland & Sussex, 1985). This study found that 73% of suicides clustered in four multigenerational families, which encompassed only 16% of the total population. The study's focus on the isolated Old Order Amish community, however, greatly limits the generalizability of its findings.

The current urgency to reduce suicide rates and develop well-performing predictive models of suicide in US populations (Gordon, Avenevoli, & Pearson, 2020) necessitates the estimation of specific measures of how suicide aggregates in American families. The US state of Utah is unique in the availability of data resources that make feasible a population-based analysis of the familial aggregation of suicide. The Utah Population Database (UPDB) is a repository for multiple population-wide sources of biomedical-related information and includes information on multi-generational pedigrees (Smith & Fraser, 2018). Using multigenerational data from the UPDB linked to mortality information, the current study aimed to determine the familial aggregation of suicide in Utah in first- through fifth-degree relatives. To expand upon prior work, we also examined if and how the familial risk of suicide varies in first- through fifth-degree relatives based on sex and age of the suicide proband and sex of the proband's relative. Analyses were conducted using a unified modeling approach (Lee, Rebora, Valsecchi, Czene, & Reilly, 2013), which minimizes the total number of models needed to test kinship and interaction effects while simultaneously maximizing statistical efficiency. Finally, the attributable risk and the population attributable fraction of the familial risk of suicide were calculated to measure its contribution on individual and population levels to suicide death in Utah.

## Methods

### Study population and sampling design

The current study used a prospective cohort design to determine the familial aggregation of suicide in relatives of suicide probands *v.* relatives of non-suicide controls. The study population was identified in the UPDB, a unique, multi-source comprehensive data resource containing genealogical, demographic, and vital records on over 11 million current and previous Utah residents (Smith & Fraser, 2018). The suicide group included all Utah suicides from 1904 to 2014 who were 10 years or older at time of death (six suicides were excluded for age <10 at time of death) based on a suicide manner of death indication on a death certificate. For deaths from 1957 to 2014, International Classification of Diseases (ICD) cause-of-death coding was used to identify additional suicides including ICD-6 codes E970-E979, ICD-7 codes E963 and E970-E979, ICD-8 and 9 codes E950-D959, and ICD-10 codes X60-X84 and U03. For deaths from 1904 to 1956, identification of suicide was based on UPDB translation of death certificate causes-of-death text into corresponding ICD-10 codes using the 2000 Mortality Medical Data System developed by the US Department of Health and Human Services (Lu, 2003; MMDS, 2015). Covariate information obtained for suicide probands included sex, birth year, and death year. Each proband was matched to five non-suicide controls based on sex and birth year using at-risk sampling whereby a potential control had to be alive at the time of the suicide proband's death but could have died by suicide at a later time.

The UPDB maintains extensive, multi-generational genealogies with founding family members belonging to Utah's original European settlers who migrated to present-day Utah beginning in the mid-19th century. Data from Utah state vital records including birth, death, and divorce certificates are used to construct *de novo* genealogies, and follow-up information is acquired through regular linkage to state driver license information, records from the Centers of Medicaid and Medicare, and to the Social Security Death Index. The largest families in UPDB include up to 18 generations. First (i.e. siblings, parents, children), second (i.e. grandparents, grandchildren, aunts, uncles, nieces, nephews), third (i.e. great-grandparents, great-grandchild, first cousins, great-uncles, great-aunts, great-nieces, great-nephews), and fifth (i.e. second cousins) degree relatives of suicide probands and matched controls were identified. Additional information obtained for relatives included their sex, birth year, last known year residing in Utah or death year, and manner of death.

**Fig. 1.**
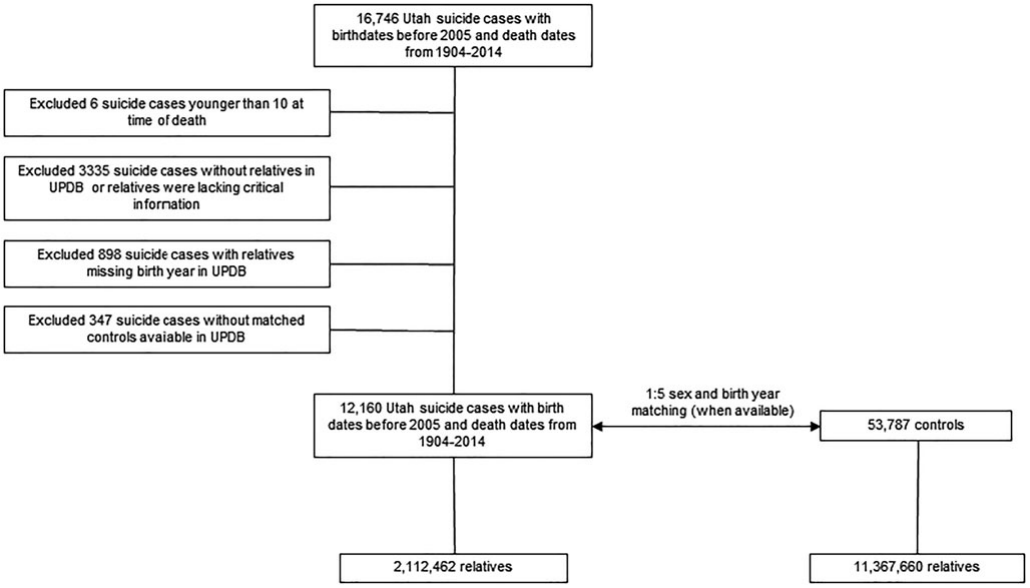
Flow diagram describing the Utah suicide proband, control, and relative cohorts used in the study.

Suicide probands were excluded from the analysis if they did not link to known relatives or all relatives were missing critical information (e.g. sex, no follow-up or death information, *N* = 3335), all of their identified relatives were missing birth year information (*N* = 898), or if appropriate controls were not available (*N* = 347) (Fig. 1). Family clusters were identified to take into account the non-independent, correlated structure of the data. A single family cluster included the combined set of all first through fifth-degree relatives of each proband and their matched controls (online Supplementary Fig. S1).

### Statistical methods

#### Familial risk of suicide

The analysis was conducted in the relatives of the suicide probands and controls whereby the exposure was the suicide proband or the matched control. The familial aggregation of suicide was quantified in a unified modeling framework (Lee et al., 2013) by hazard ratios (HR) from an unsupervised Cox regression model, which measured the incidence of suicide in relatives of suicide probands *v.* controls. Age was used as the underlying time-scale. To account for left censoring due to missing manner of death information, relatives of suicide cases and controls born before 1904 entered the model starting with their age in 1904. All relatives were followed until suicide, death by non-suicide, loss to UPDB follow-up (e.g. migration out of Utah) or 31 December 2014, whichever came first. Individuals populating the suicide proband/control and relative (analysis) cohorts could have contributed to the analysis in multiple ways. First, a small proportion of the control group died of suicide at a later date thereby serving as both a suicide proband and control (*N* = 245). Second, individual relatives may have belonged to more than one family cluster. A robust sandwich variance estimator (Wei, Lin, & Weissfeld, 1989) was used to account for dependence among individual family members. Additional covariates included in the model were characteristics of the proband such as sex and characteristics of the relatives such as birth year, sex, and type of relationship to the proband (e.g. sibling, aunt, first cousin).

The hazard of suicide in specific kinships (e.g. children–parents, grandchildren–grandparents) were estimated using two-way interactions between exposure (i.e. relative was exposed to a suicide or control proband) and relative's relationship to the proband. Similarly, four-way interactions that included exposure, proband's sex, relative's sex, and relative's relationship to the proband were used to test for moderation of the familial suicide HR based on sex of the suicide proband or relative. To investigate age effects, the unified Cox regression model was stratified by suicide probands <25 years or ⩾25 years at time of death. This age was selected as prior Utah-based work examining clinical correlates of youth suicide examined suicide through age 25 (Keeshin, Gray, Zhang, Presson, & Coon, 2018). Sensitivity analyses were conducted to examine the robustness of study findings. First, to investigate the potential influence of families with extremely high aggregation of suicide among first-degree relatives, families with (1) three or (2) four or more siblings who died by suicide were removed from the analysis. Next, to investigate the influence of alternative age cut-offs, additional Cox regression models were stratified by suicide probands (1) ⩽18 years or >18 years, and (2) <41 years or ⩾41 years, which is mean age of suicide in Utah (Table 1). Finally, to test the potential influence of using at-risk sampling whereby a matched control could have died by suicide at a later date, additional age-stratified (<25 *v.* ⩾25 years) Cox regression models were formulated with the 245 matched controls who later died by suicide removed from the analysis.

**Table 1.** Demographic characteristics of Utah suicides, matched controls and relatives of suicides and matched controls

	Suicide probands				Non-suicide probands			
	Probands (*N* = 12 160)		Relatives (*N* = 2 112 462)		Controls (*N* = 53 787)		Relatives (*N* = 11 367 660)	
Characteristic	*N*	%	*N*	%	*N*	%	*N*	%
Sex								
Female	2496	20.5	1 030 090	48.8	10 920	20.3	5 538 828	48.7
Male	9664	79.5	1 082 372	51.2	42 867	79.7	5 828 832	51.3
Age at time of suicide, mean (s.d.), years	41.74 (17.3)		52.41 (26.1)		41.53 (17.5)		52.40 (26.2)	
Age range, years								
<34	4513	37.1	589 473	27.9	20 479	38.1	3 207 281	28.2
34–64	6251	51.4	700 050	33.1	27 084	50.4	3 717 959	32.7
>64	1396	11.5	822 939	39.0	6224	11.6	4 442 420	39.1
Birth range, years								
<1850	27	0.2	33 397	1.6	112	0.2	179 490	1.6
1850–1875	224	1.8	79 042	3.8	1018	1.9	418 489	3.7
1876–1900	801	6.6	231 060	10.9	3732	6.9	1 244 319	11.0
1901–1925	1916	15.8	493 874	23.4	8776	16.3	2 653 233	23.3
1926–1950	2778	22.9	520 179	24.6	11 997	22.3	2 759 547	24.3
1951–1975	4524	37.2	464 998	22.0	19 319	35.9	2 491 502	21.9
1976–2000	1887	15.5	252 192	11.9	8818	16.4	1 391 466	12.2
>2000	3	<0.1	37 720	1.8	15	<0.1	229 614	2.0

*N*, number; s.d., standard deviation.

#### Attributable risk and population attributable fraction

The attributable risk of suicide is a calculation of the percent of suicides among relatives of suicide probands that is attributable to familial risk. The population attributable fraction is a measure of the percent of Utah suicides that is attributed to familial risk. Age-adjusted attributable and population attributable risk fractions were estimated using the indirect standardization approach according to the STDRATE procedure in SAS (Yuan, 2013). The calculation of attributable risk and the population attributable fraction included all 12 160 suicide deaths used in the main analysis.

Analyses were conducted in SAS version 9.4 (SAS Institute Inc., Cary, NC, USA) and a Type I error was set at 0.05. Institutional Review Board approval was obtained from the University of Utah and the Utah Resource for Genetic and Epidemiologic Research. STROBE reporting guidelines were followed in preparing this study.

## Results

### Sample characteristics

The final sample included 12 160 Utah suicide probands identified in UPDB born before 2005 with death dates from 1904 to 2014; suicide probands were matched to 53 787 non-suicide probands (Fig. 1). Relatives of suicide and non-suicide probands included 2 112 462 and 11 367 660 first through fifth-degree relatives (includes duplicate individuals that belong to more than one suicide or non-suicide family cluster), respectively. Table 1 summarizes the demographic characteristics of the suicide, matched control, and relatives groups.

### Familial risk of suicide in first through fifth-degree relatives

A significantly heightened familial risk of suicide was measured in all first through fifth-degree relatives (Table 2). The overall hazard of suicide was 1.26 [95% confidence intervals (CI) 1.22–1.30] among all first through fifth-degree relatives. First-degree relatives of suicide probands were at more than three times the risk of suicide compared with first-degree relatives of non-suicide probands (HR 3.45; 95% CI 3.12–3.82). The HR of suicide was 3.20 (95% CI 2.71–3.77) in parents, 3.46 (95% CI 2.98–4.03) in children, and 3.67 (95% CI 3.18–4.24) in siblings of suicide probands *v.* non-suicide probands. The familial risk of suicide in second- through fifth-degree relatives displayed a decreasing dose–response relationship with the HRs ranging from 1.85 (95% CI 1.67–2.06) in nieces/nephews to 1.07 (95% CI 1.02–1.12) in second cousins of suicide probands. A closer examination of parent–child dyads in which both a parent and a child died by suicide showed that in 75% of these cases, the parent died first. However, the mean number of years between parent–child suicides is considerably shorter when the child dies first (9.9 *v.* 19.2 years; online Supplementary Table S1).

**Table 2.** Familial risk of suicide in first-, second-, third-, and fifth-degree relatives of suicides and matched controls in Utah^a^

	Suicide probands		Non-suicide probands			
Relation to proband	*N* suicides	*N* relatives	*N* suicides	*N* relatives	Hazard ratio	95% CI
First-degree relatives						
**Overall**	**1134**	**73 450**	**1760**	**390 822**	**3**.**45**	**3.12–3.82**
Parent	275	20 977	399	95 246	3.20	2.71–3.77
Child	272	18 234	510	121 435	3.46	2.98–4.03
Sibling	587	34 239	851	174 141	3.67	3.18–4.24
Second-degree relatives						
**Overall**	**1483**	**198 370**	**4473**	**1 071 859**	**1**.**79**	**1.65–1.94**
Grandparent	162	30 482	453	148 869	1.75	1.45–2.10
Grandchild	162	23 844	639	159 523	1.70	1.43–2.02
Uncle or aunt	574	73 381	1645	373 334	1.78	1.60–1.99
Niece or nephew	585	70 663	1736	390 133	1.85	1.67–2.06
Third-degree relatives						
**Overall**	**2768**	**563 932**	**11 668**	**3 025 042**	**1**.**27**	**1.20–1.35**
Great grandparent	107	47 135	436	237 124	1.20	0.95–1.53
Great grandchild	111	25 565	561	175 520	1.35	1.09–1.67
Great uncle or aunt	628	147 999	2403	737 251	1.29	1.17–1.43
Great niece or nephew	646	129 651	2714	721 352	1.30	1.18–1.44
First cousin	1276	213 582	5554	1 153 795	1.25	1.15–1.36
Fifth-degree relatives						
**Overall**^b^	**6767**	**1 276 710**	**34 179**	**6 879 937**	**1**.**07**	**1.02–1.12**

*N*, number.

a Model was adjusted for relative's birth year and sex and proband's sex.

b All fifth-degree relatives included in this study were second cousins.

### Differences by sex

**Fig. 2.**
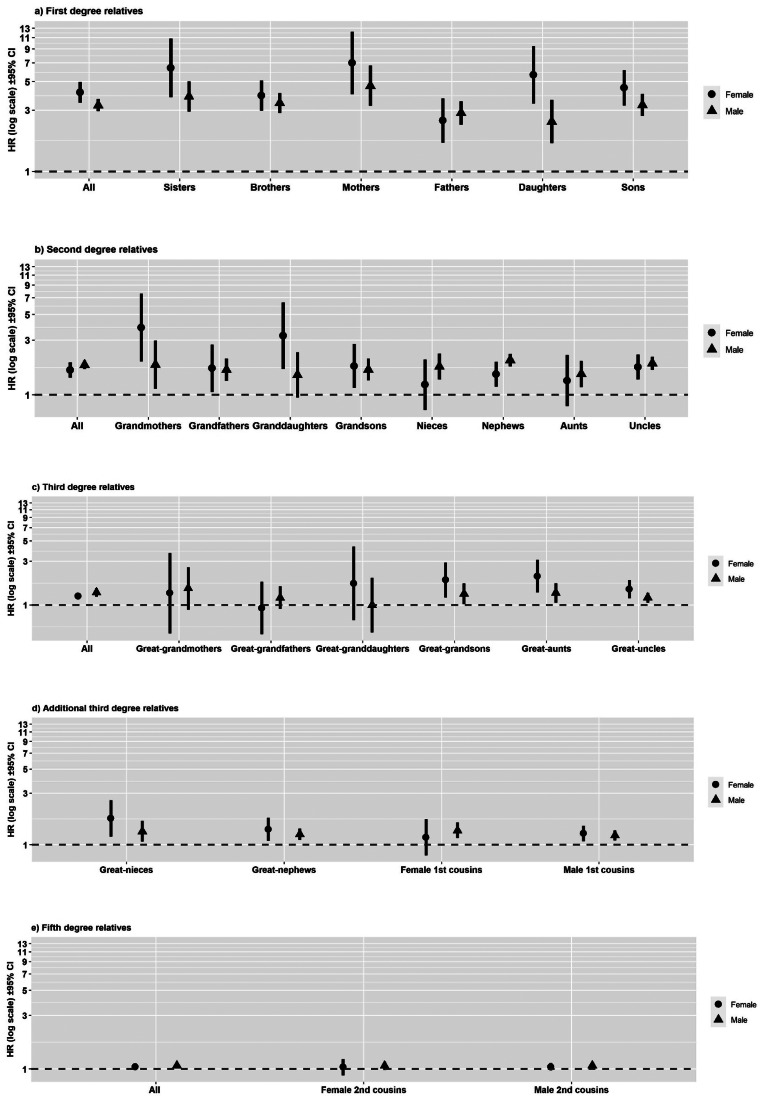
Suicide hazard ratios (HR) ± 95% confidence intervals (*y*-axis) in relatives of suicide probands *v.* controls in first- through fifth-degree relatives stratified by suicide proband's sex. Relative of suicide proband is listed on the *x*-axis. The models were adjusted for relative's sex and birth year and proband's sex.

The familial risks of suicide based on sex of the proband and relative in specific kinships are shown in Fig. 2. Among first-degree relatives, the HR of suicide was 6.99 (95% CI 3.99–12.25) in mothers of female suicide probands compared with mothers of non-suicide female probands. Female probands were also associated with high suicide risks in sisters (HR 6.39; 95% CI 3.78–10.82), daughters (HR 5.65; 95% CI 3.38–9.44), sons (HR 4.48; 95% CI 3.26–6.14), grandmothers (HR 3.84; 95% CI 1.95–7.58), and granddaughters (HR 3.27; 95% CI 1.68–6.35).

Differences in the familial risk of suicide according to the proband or relative's sex persisted out to third-degree relatives in some specific kinships. Among great-aunts, the HR associated with the suicide of a great-niece was 2.06 (95% CI 1.37–3.09) compared with an HR of 1.35 (95% CI 1.06–1.73) associated with the suicide of a great-nephew. In contrast, in many kinships where the proband was male, the familial risk of suicide did not differ between male and female relatives. For example, the HR of suicide was similar for brothers (HR 3.40; 95% CI 2.85–4.07) and sisters (HR 3.83; 95% CI 2.92–5.03) of male suicide probands.

### Differences by age and age sensitivity analysis

**Fig. 3.**
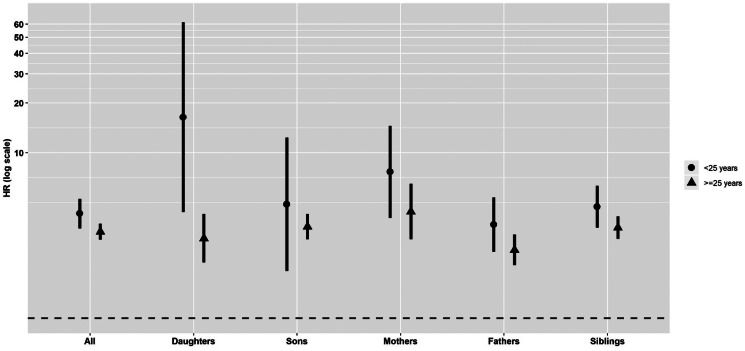
Suicide hazard ratios (HR) ± 95% confidence intervals (*y*-axis) in first-degree relatives of suicide probands *v.* controls stratified by <25 *v.* ⩾25 years of age at time of death. Relative of suicide proband is listed on the *x*-axis. The models were adjusted for relative's sex and birth year and proband's sex.

Figure 3 displays the hazard ratios in first-degree relatives of suicide probands *v.* matched controls stratified by age (<25 *v.* ⩾25 years). The suicide HR was 4.29 (95% CI 3.49–5.26) for all first-degree relatives of suicide probands who were <25 years at death compared with 3.33 (CI 2.98–3.72) for all first-degree relatives of suicide probands who were ⩾25 years at death. Higher HR estimates for relatives of younger suicide probands *v.* relatives of older suicide probands persisted across all specific first-degree kinships as well as for most second through fifth-degree kinships, although the risk differences for relatives of younger *v.* older suicide probands narrows as the relationship becomes more distant (online Supplementary Fig. S2). The highest familial risk of suicide reported in this study was measured among daughters of a parent who died by suicide before age 25 (HR 16.36; 95% CI 4.36–61.44). Using 18 years as the age cut-off for youth suicide did not impact study findings concerning age effects (online Supplementary Table S2). However, data sparseness when using age 18 years as the cut-off prevented HR estimation in some first-degree kinships. The effects of age on suicide risk appeared to disappear when stratifying suicide deaths by the mean age of suicide (41 years) in Utah (online Supplementary Table S3). Study results for the <25 *v.* ⩾25 years age-stratified models were robust to the removal of matched controls who later died by suicide (online Supplementary Table S4).

### Sensitivity analysis, attributable risk, and population attributable risk fraction

Forty-three families with three or more siblings who died by suicide were removed from the analysis and four families with four or more siblings who died by suicide were removed from the analysis. The removal of families with high aggregation of suicide among siblings did not substantively alter the general study findings (online Supplementary Table S5). Finally, the attributable risk of familial suicide was 0.20 (95% CI 0.19–0.22) and the population attributable risk fraction of familial suicide was 0.04 (95% CI 0.035–0.042).

## Discussion

The current study represents the first total population-wide investigation of the familial risk of suicide in the USA. Our study included all suicides occurring in a 110-year period in Utah as well as 13.5 million relatives of suicide probands and control. The identification of first- through fifth-degree relatives of suicide probands and controls, born as far back as the 1800s, allowed us to thoroughly examine the familial risk of suicide by degree of genetic relationship.

We found that significant familial liabilities of suicide extend out to fifth-degree relatives and that the sex and age of a suicide proband impacts the suicide risk in his/her relatives. Study findings highlight the importance of acquiring information on an individual's family history of suicide, including the age and sex of the suicide decedent, to prevent suicide in potentially high-risk individuals.

Overall, the risk of suicide was 26% higher among all combined first- through fifth-degree relatives of suicide cases compared with controls. While prior studies have not considered up to fifth-degree relationships, our Utah-based estimates for some first through third-degree kinships are consistent with familial liability estimates reported in similarly designed studies conducted in Northwestern European populations. The current study and a Danish-based study (Qin et al., 2003) both report an overall 3.5-fold increase in the familial risk of suicide in first-degree relatives of suicide probands. A Swedish-based study (Tidemalm et al., 2011) identified relative risks of 3.1 in siblings (*v.* 3.7 in Utah), 1.6 in nieces/nephews (*v*. 1.9 in Utah), and 1.5 in first cousins (*v*. 1.3 in Utah) of suicide probands. In contrast, two population-based studies conducted in Denmark (Qin et al., 2002) and Sweden (Runeson & Åsberg, 2003) identified a more modest two-fold increase in the familial risk of suicide in first-degree relatives.

The opportunity to compare Utah-specific estimates with Northwestern European-based estimates may be particularly relevant given the ancestry of Utah's suicides. While the majority of Utah suicide cases self-identify as White, non-Hispanic (see Limitations section), a recent molecular analysis, allowing for a more refined look at ancestry, determined that 80% of a population-based sample of Utah suicide decedents from 1996 to 2017 (*N* = 4379) were of majority Northwestern European ancestry (Docherty et al., 2020). Interestingly, at 21.2 per 100 000 persons (AFSP, 2019), Utah's age-adjusted suicide rate is considerably higher than those observed in Northwestern Europe [e.g. the age-adjusted suicide rate is 11.7 per 100 000 persons in Sweden (World Health Organization, 2019)]. While prior work demonstrates consistency in relative suicide rates among European countries over time (Hansen & Pritchard, 2008), stark differences in age-adjusted suicide rates between Utah and Northwestern Europe suggest that a unique set of factors, beyond ancestral similarities, influence suicide risk differences between locations.

Suicide aggregates in families due to a combination of genetic, shared environmental or gene–environment interactions and the current study was not designed to tease apart the unique contribution of each of these factors. In consideration of genetic contributors, our discovery of significantly elevated familial suicide risks in second- through fifth-degree kinships is an important and novel contribution. The detection of significant familial clustering of suicide well beyond the nuclear family may be evidence of genetic sharing within Utah's extended families, as more distantly related relatives are less likely to share environmental exposures. A considerable genetic contribution to suicide supports findings from prior adoption (Kendler et al., 2020; Petersen et al., 2013; Schulsinger et al., 1979; von Borczyskowski et al., 2011) and twin (Juel-Nielsen & Videbech, 1970; Pedersen & Fiske, 2010; Roy et al., 1991) studies. The specific genes implicated in suicide, however, remain unclear. While over 200 genes are associated with suicidal behaviors (Lutz, Mechawar, & Turecki, 2017), many of these gene findings require replication. Further, the majority of gene-focused suicide studies have been conducted in individuals exhibiting suicidal behavior and not in suicide decedents (Coon et al., 2020) (but see Coon et al., 2020; Darlington et al., 2014; Docherty et al., 2020; Otsuka et al., 2019; Tombáca et al., 2017). To better understand suicide's genetic architecture, additional work is needed in suicide death cohorts.

Several environmental contributors to suicide are known to be transmitted through families and may impact study findings, especially among close kinships. Increased suicide risk during youth and adolescence has been linked to exposure to early-life adversity from parental neglect, substance abuse, criminality (Björkenstam, Hjern, Björkenstam, & Kosidou, 2018; Björkenstam, Kosidou, & Björkenstam, 2017; Brent et al., 1994), and interpersonal violence (Rajalin, Hirvikoski, & Jokinen, 2013). Epigenetic modification following exposure to early-life adversity of genes involved in pathways underlying suicide including neural plasticity, neuroprotection, stress, and cognition provides evidence of potential biological effects from such exposures (Turecki & Brent, 2016). Beyond youth and adolescent suicide, a family history of psychiatric conditions and hospitalization for psychiatric conditions among first-degree relatives has been shown to be linked to an increased risk of suicide across the lifespan (Agerbo et al., 2002; Qin et al., 2002, 2003). In contrast, little is currently known about how a family history of early-life adversity and psychopathology influences the risk of suicide in more distantly related relatives (e.g. beyond first-degree relationships).

Our findings of heightened familial risks of suicide in extended family members may be evidence of gene–environment interactions. While distant relatives are less likely than close relatives to share environmental exposures, the environmental factors they do share are more likely to be those that are widespread across a population (Amundadottir et al., 2004). Suicide population attributable fraction estimates attest to the important contribution of widespread, population-level environmental factors to risk. For example, the attributable risks of suicide associated with being single range from 10.3% (Qin et al., 2003) to 25.6% (Mortensen, Agerbo, Erikson, Qin, & Westergaard-Nielsen, 2000).

We found that sex and age influence the familial risk of suicide. Although suicide is more common in males, our work supports prior research demonstrating especially heightened liabilities of suicide in first-degree relatives of female (Cheng et al., 2014; Garssen et al., 2011; Petersen et al., 2013; Qin et al., 2003; Qin & Mortensen, 2003) and younger (Garssen et al., 2011; Qin & Mortensen, 2003) suicide decedents. Our findings also indicate that female and youth transmission of risk extends beyond the nuclear family. The heritability of suicide mortality has been hypothesized to be higher in women than men (Pedersen & Fiske, 2010), which might in-part explain the high risks of suicide in female-specific kinships (e.g. mother-daughters, sisters). Alternatively, the loss of a mother may represent the disappearance of a critical source of support and caregiving. Such loss may be especially heightened among daughters as parental attachment and the transmission of learned behaviors to children appears to be more strongly linked to their same-sex parent (Diener, Isabella, Behunin, & Wong, 2008). In addition, psychopathology is a strong risk factor for suicide (Arsenault-Lapierre, Kim, & Turecki, 2004; Qin et al., 2002) with some forms of psychopathology (i.e. major depressive disorder) occurring more frequently in women (Salk, Hyde, & Abramson, 2017; Oquendo et al., 2001). Although suicide and psychopathology risk both cluster in families, their transmission, despite some overlap, has been shown to be distinct (Brent, Bridge, Johnson, & Connolly, 1996; Egeland & Sussex, 1985; Qin et al., 2003). It is not known, however, if the familial transmission of suicide and psychopathology differs by sex and is more likely to occur among women. In contrast, it is important to note that the low absolute suicide rate in females in Utah could result in relatively high hazard ratios. In terms of age effects, an inverse relationship has been observed between early age-of-onset and familial risk for a variety of medical and psychiatric conditions (Gillespie, Gale, & Bingley, 2002; Kendler, Gatz, Gardner, & Pedersen, 2005; Kharazmi, Fallah, Sundquist, & Memminki, 2012; Nestadt et al., 2000).

Limitations of the current study are important to recognize. First, although the models were adjusted for relative's sex and birth year and proband's sex, they were not adjusted for additional confounders including socioeconomic status, urbanicity, and history of mental illness and trauma. Prior work indicates that familial suicide risk estimates are robust to adjustment by additional covariates, especially among first-degree relatives who likely share potential confounders, however their inclusion has been shown to slightly attenuate effect sizes (Qin et al., 2002, 2003). The generalizability of study findings to the broader USA is unknown and may, in particular, be influenced by the racial and ethnic composition of Utah's suicide population. Information in the UPDB indicates that 95.2% of the suicide deaths since 1904 self-identified as White, non-Hispanic. Of note, the 3335 suicide deaths excluded from the study due to a lack of relatives in UPDB, who might be expected to be more recent in-migrants to Utah and therefore more racially and ethnically diverse, were of similar race and ethnicity (92.3% White, non-Hispanic). Although racially and ethnically homogeneous, Utah's age-adjusted suicide rates are among the highest in the USA (AFSP, 2019) suggesting that Utah-specific discoveries may apply to other high suicide risk populations in the USA with similar demographic compositions. Finally, the current study relied on death certificate determination of suicide, which is susceptible to possible misclassification of suicide deaths as undetermined or accidental (Mohler & Earls, 2001; Ohberg & Lonnqvist, 1998; Rosenberg et al., 1988). In particular, the majority of Utah's population are members of the Church of Jesus Christ of Latter Day Saints (LDS), and evidence suggests that suicide misclassification may be more likely in areas with strong single religion identities (Prichard & Hansen, 2015). Utah-based work, however, reports that highly active male members of the LDS church are at a reduced risk of suicide relative to their less active and non-LDS peers (Hilton, Fellingham, & Lyon, 2002). Consistency in the method of death determination in Utah is further assisted by the use of a single, centralized Office of the Medical Examiner. Any death misclassification is likely to result in a conservative determination of suicide with the associated bias attenuating effect estimates toward the null.

Despite these limitations, our study has a number of strengths. The UPDB provides access to unprecedented resources for conducting population-wide analyses; a similar study of this magnitude is not currently feasible elsewhere in the USA. The depth of genealogical and death certificate data maintained in UPDB is more extensive than what is available in similar, primarily European health registries. We used a unified modeling approach, which minimized the formulation of multiple models thereby improving effect estimate precision. Further, our modeling approach accounted for participant clustering within multiple families reducing the risk of selection bias (Bai, Sherman, Khoury, & Flanders, 2000).

In conclusion, we found that the familial liability of suicide extends from first- through fifth-degree relatives in Utah with the magnitude of risk in relatives varying based on their sex and the sex and age of the suicide proband. The attributable risk of familial suicide was estimated to be 20% suggesting that our findings have important implications for suicide prevention on an individual-level and within a family. In particular, our study indicates an increased intensity of interventions for suicidal young adults and women with a strong family history of suicide. Further attention to this approach to suicide prevention may be especially warranted in the USA given recent increases in suicide rates among young adults (Miron, Yu, Wilf-Miron, & Kohane, 2019), especially females (Ruch et al., 2019).

## Supplementary material

For supplementary material accompanying this paper visit https://doi.org/10.1017/S0033291721003020. click here to view supplementary material
