# Supplementary material for: A population-wide analysis of the familial risk of suicide in Utah, USA
Source: Psychol Med. 2021 Aug 10;53(4):1448–57. doi: 10.1017/S0033291721003020 (PMC10009406; doi:10.1017/S0033291721003020)
Supplement: Supplementary file 1 [file S0033291721003020sup001.docx]

**SUPPLEMENTAL MATERIALS**

| **Supplementary Table S1. Frequency and description of parent-child suicides in Utah from 1904-2014.** | | | | | | | | |
| --- | --- | --- | --- | --- | --- | --- | --- | --- |
|  |  | **Proband is the parent, relative is the child** | | |  | **Proband is the child, relative is the parent** | | |
| **Kinship** | **N Kinship suicides** | **N parent dies first** | **% parent dies first** | **Mean (SD) years between death** |  | **N child dies first** | **% child dies first** | **Mean (SD) years between death** |
| Mother-Daughter | 21 | 14 | 67% | 20.5 (9.3) |  | 7 | 33% | 9.4 (10.8) |
| Mother-Son | 57 | 37 | 65% | 12.5 (9.9) |  | 20 | 35% | 9.1 (7.7) |
| Father-Son | 158 | 124 | 78% | 20.8 (14.8) |  | 34 | 22% | 11.4 (11.0) |
| Father-Daughter | 33 | 25 | 76% | 20.4 (14.7) |  | 8 | 24% | 5.7 (3.9) |
| All | 269 | 200 | 75% | 19.2 (13.9) |  | 69 | 25% | 9.9 (9.5) |

| **Supplementary Table S2. Familial risk of suicide in first, second, third, and fifth degree relatives of suicides and matched controls in Utah stratified by ≤18 years versus >18 years**^a^ | | | | | | | |
| --- | --- | --- | --- | --- | --- | --- | --- |
|  | **Suicides** | |  | **Matched controls** | |  |  |
| Relation to proband | **N suicides** | **N relatives** |  | **N suicides** | **N relatives** | **Hazard ratio** | **95% CI** |
| ≤18 years | | | | | | | |
| **Overall 1^st^ degree relatives** | **53** | **3163** |  | **78** | **19390** | **3.95** | **2.77 - 5.63** |
| Mother | 10 | 761 |  | 3 | 3567 | 15.77 | 4.33 - 57.49 |
| Father | 16 | 727 |  | 24 | 3376 | 3.08 | 1.69 - 5.64 |
| Daughter | 0 | 4 |  | 3 | 1610 | 0 | 0 - 0 |
| Son | 0 | 3 |  | 9 | 1757 | 0 | 0 - 0 |
| Sibling | 27 | 1668 |  | 39 | 9080 | 4.35 | 2.65 - 7.15 |
| **Overall 2^nd^ degree relatives** | **54** | **7379** |  | **203** | **42403** | **1.48** | **1.08 - 2.02** |
| Grandchildren | 0 | 6 |  | 10 | 1391 | 0 | 0-0 |
| Grandparent | 14 | 1979 |  | 43 | 9538 | 1.56 | 0.84 - 2.89 |
| Nieces/Nephew | 6 | 1866 |  | 46 | 11864 | 0.88 | 0.37 - 2.08 |
| Aunt/Uncle | 34 | 3528 |  | 104 | 18610 | 1.70 | 1.14 - 2.53 |
| **Overall 3^rd^ degree relatives** | **122** | **21846** |  | **452** | **122840** | **1.49** | **1.19 - 1.86** |
| Great Grand children | 0 | 0 |  | 7 | 2212 | 0 | 0 - 0 |
| Great Grand parent | 11 | 3109 |  | 38 | 15653 | 1.40 | 0.71 - 2.74 |
| Great Nieces/nephew | 8 | 2156 |  | 36 | 14518 | 1.32 | 0.48 - 3.67 |
| Great Aunts/uncle | 44 | 7724 |  | 157 | 40993 | 1.49 | 1.03 - 2.15 |
| First cousin | 59 | 8857 |  | 214 | 49464 | 1.56 | 1.15 - 2.12 |
| **Overall 5th degree relatives**^b^ | **265** | **51727** |  | **1304** | **297332** | **1.24** | **1.07 - 1.44** |
| >18 years | | | | | | | |
| **Overall 1^st^ degree relatives** | **1081** | **70287** |  | **1682** | **371432** | **3.43** | **3.09 - 3.81** |
| Mother | 69 | 9848 |  | 68 | 44550 | 4.65 | 3.28 - 6.58 |
| Father | 180 | 9641 |  | 304 | 43753 | 2.76 | 2.27 - 3.36 |
| Daughter | 55 | 8800 |  | 109 | 57386 | 3.19 | 2.29 - 4.45 |
| Son | 217 | 9427 |  | 389 | 60682 | 3.56 | 2.99 - 4.23 |
| Sibling | 560 | 32571 |  | 812 | 165061 | 3.65 | 3.15 - 4.22 |
| **Overall 2^nd^ degree relatives** | **1429** | **190991** |  | **4270** | **1029456** | **1.80** | **1.66 - 1.96** |
| Grandchildren | 162 | 23838 |  | 629 | 157132 | 1.70 | 1.43 - 2.03 |
| Grandparent | 148 | 28503 |  | 410 | 139331 | 1.76 | 1.45 - 2.15 |
| Nieces/Nephew | 579 | 68797 |  | 1690 | 378269 | 1.88 | 1.69 - 2.08 |
| Aunt/Uncle | 540 | 69853 |  | 1541 | 354724 | 1.79 | 1.59 - 2.00 |
| **Overall 3^rd^ degree relatives** | **2646** | **542086** |  | **11216** | **2902202** | **1.269** | **1.19 - 1.34** |
| Great Grandchildren | 111 | 25565 |  | 554 | 173308 | 1.36 | 1.09 - 1.68 |
| Great Grandparent | 96 | 44026 |  | 398 | 221471 | 1.18 | 0.92 - 1.53 |
| Great Nieces or Nephew | 638 | 127495 |  | 2678 | 706834 | 1.30 | 1.18 - 1.44 |
| Great Aunts or Uncle | 584 | 140275 |  | 2246 | 696258 | 1.28 | 1.15 - 1.42 |
| First Cousin | 1217 | 204725 |  | 5340 | 1104331 | 1.24 | 1.13 - 1.35 |
| **Overall 5th degree relatives^b^** | **6502** | **1224983** |  | **32875** | **6582605** | **1.064** | **1.02 - 1.11** |

| **Supplementary Table S3. Familial risk of suicide in first, second, third, and fifth degree relatives of suicides and matched controls in Utah stratified by <41 years versus ≥41 years**^a^ | | | | | | | |
| --- | --- | --- | --- | --- | --- | --- | --- |
|  | **Suicides** | |  | **Matched controls** | | |  |
| Relation to proband | **N suicides** | **N relatives** |  | **N suicides** | **N relatives** | **Hazard ratio** | **95% CI** |
| <41 years | | | | | | | |
| **Overall 1^st^ degree relatives** | 509 | 31653 |  | 814 | 175608 | 3.43 | 3.01-3.91 |
| Mother | 22 | 4911 |  | 27 | 22385 | 3.73 | 2.10- 6.65 |
| Father | 84 | 4854 |  | 124 | 22166 | 3.14 | 2.33 - 4.23 |
| Daughter | 40 | 6419 |  | 73 | 36774 | 3.08 | 2.08 - 4.56 |
| Son | 175 | 6899 |  | 255 | 38935 | 3.92 | 3.21 - 4.78 |
| Sibling | 283 | 15525 |  | 384 | 79187 | 3.96 | 3.29 - 4.76 |
| **Overall 2^nd^ degree relatives** | 605 | 77733 |  | 1848 | 425916 | 1.77 | 1.58 - 1.98 |
| Grandchildren | 21 | 3638 |  | 147 | 39011 | 1.75 | 1.1 - 2.8 |
| Grandparent | 94 | 15624 |  | 312 | 76075 | 1.48 | 1.17 - 1.86 |
| Nieces/Nephew | 192 | 25843 |  | 530 | 144171 | 2.03 | 1.7 - 2.42 |
| Aunt/Uncle | 298 | 32628 |  | 859 | 166659 | 1.77 | 1.53 - 2.03 |
| **Overall 3^rd^ degree relatives** | 1162 | 224269 |  | 4720 | 1217238 | 1.33 | 1.23 - 1.44 |
| Great Grandchildren | 9 | 2856 |  | 80 | 34840 | 1.31 | 0.73 - 2.38 |
| Great Grandparent | 71 | 24800 |  | 274 | 125808 | 1.28 | 0.97 - 1.68 |
| Great Nieces or Nephew | 156 | 36435 |  | 607 | 209830 | 1.49 | 1.24 - 1.79 |
| Great Aunts or Uncle | 371 | 71623 |  | 1428 | 367293 | 1.32 | 1.16 - 1.50 |
| First Cousin | 555 | 88555 |  | 2331 | 479467 | 1.30 | 1.15 - 1.46 |
| **Overall 5th degree relatives^b^** | 2850 | 541737 |  | 14648 | 2961884 | 1.08 | 1.02 - 1.14 |
| ≥41 years | | | | | | | |
| **Overall 1^st^ degree relatives** | 625 | 41797 |  | 946 | 215214 | 3.48 | 3.04 - 3.97 |
| Mother | 57 | 5698 |  | 44 | 25732 | 5.95 | 3.99 - 8.87 |
| Father | 112 | 5514 |  | 204 | 24963 | 2.57 | 2.04 - 3.25 |
| Daughter | 15 | 2385 |  | 39 | 22222 | 3.88 | 2.07 - 7.25 |
| Son | 42 | 2531 |  | 143 | 23504 | 2.87 | 2.03 - 4.05 |
| Sibling | 304 | 18714 |  | 467 | 94954 | 3.45 | 2.84 - 4.18 |
| **Overall 2^nd^ degree relatives** | 878 | 120637 |  | 2625 | 645943 | 1.80 | 1.63 - 1.99 |
| Grandchildren | 141 | 20206 |  | 492 | 120512 | 1.71 | 1.41 - 2.06 |
| Grandparent | 68 | 14858 |  | 141 | 72794 | 2.34 | 1.71 - 3.19 |
| Nieces/Nephew | 393 | 44820 |  | 1206 | 245962 | 1.78 | 1.57 - 2.02 |
| Aunt/Uncle | 276 | 40753 |  | 786 | 206675 | 1.80 | 1.54 - 2.11 |
| **Overall 3^rd^ degree relatives** | 1606 | 339663 |  | 6948 | 1807804 | 1.24 | 1.15 - 1.32 |
| Great Grandchildren | 102 | 22709 |  | 481 | 140680 | 1.34 | 1.06 - 1.68 |
| Great Grandparent | 36 | 22335 |  | 162 | 111316 | 1.07 | 0.72 - 1.60 |
| Great Nieces or Nephew | 490 | 93216 |  | 2107 | 511522 | 1.25 | 1.12 - 1.40 |
| Great Aunts or Uncle | 257 | 76376 |  | 975 | 369958 | 1.25 | 1.07 - 1.45 |
| First Cousin | 721 | 125027 |  | 3223 | 674328 | 1.21 | 1.1 - 1.34 |
| **Overall 5th degree relatives^b^** | 3917 | 734973 |  | 19531 | 3918053 | 1.06 | 1.01 - 1.12 |

| **Supplementary Table S4. Familial risk of suicide in first, second, third, and fifth degree relatives of suicides and matched controls in Utah stratified by <25 years versus ≥25 years** **with 245 matched controls who later died by suicide removed**^a^ | | | | | | | |
| --- | --- | --- | --- | --- | --- | --- | --- |
|  | **Suicides** | |  | **Matched controls** | |  |  |
| Relation to proband | **N suicides** | **N relatives** |  | **N suicides** | **N relatives** | **Hazard ratio** | **95% CI** |
| <25 years | | | | | | | |
| **Overall 1^st^ degree relatives** | 175 | 9780 |  | 226 | 58098 | 4.32 | 3.52 - 5.30 |
| Mother | 25 | 2165 |  | 15 | 9958 | 7.63 | 4.01 - 14.49 |
| Father | 47 | 2077 |  | 59 | 9538 | 3.67 | 2.52 - 5.36 |
| Daughter | 3 | 150 |  | 8 | 5538 | 16.79 | 4.44 - 63.54 |
| Son | 5 | 164 |  | 35 | 6041 | 4.92 | 1.94 - 12.47 |
| Sibling | 95 | 5224 |  | 109 | 27023 | 4.78 | 3.57 - 6.41 |
| **Overall 2^nd^ degree relatives** | 213 | 24037 |  | 580 | 131942 | 1.97 | 1.66 - 2.32 |
| Grandchildren | 1 | 194 |  | 26 | 8737 | 2.64 | 0.33 - 21.08 |
| Grandparent | 40 | 5755 |  | 124 | 27552 | 1.56 | 1.09 - 2.22 |
| Nieces/Nephew | 47 | 7014 |  | 135 | 39515 | 2.04 | 1.45 - 2.88 |
| Aunt/Uncle | 125 | 11074 |  | 295 | 56138 | 2.12 | 1.71 - 2.62 |
| **Overall 3^rd^ degree relatives** | 381 | 70057 |  | 1474 | 383372 | 1.39 | 1.23 - 1.58 |
| Great Grand children | 0 | 104 |  | 18 | 7551 | 0.003 | 0.00 - 0.01 |
| Great Grand parent | 27 | 9046 |  | 105 | 45520 | 1.25 | 0.82 - 1.91 |
| Great Nieces/nephew | 31 | 8283 |  | 126 | 51291 | 1.56 | 1.01 - 2.41 |
| Great Aunts/uncle | 142 | 23954 |  | 538 | 124451 | 1.36 | 1.11 - 1.65 |
| First cousin | 181 | 28670 |  | 687 | 154559 | 1.42 | 1.18 - 1.7 |
| **Overall 5th degree relatives**^b^ | 885 | 168710 |  | 4373 | 930291 | 1.14 | 1.05 - 1.24 |
| ≥25 years | | | | | | | |
| **Overall 1^st^ degree relatives** | 944 | 61941 |  | 1494 | 323578 | 3.36 | 3.01 - 3.75 |
| Mother | 52 | 8218 |  | 54 | 37082 | 4.41 | 2.98 - 6.53 |
| Father | 147 | 8063 |  | 261 | 36523 | 2.63 | 2.12 - 3.27 |
| Daughter | 51 | 8420 |  | 100 | 52036 | 3.11 | 2.21 - 4.38 |
| Son | 209 | 9032 |  | 358 | 54883 | 3.58 | 2.99 - 4.28 |
| Sibling | 485 | 28208 |  | 721 | 143054 | 3.56 | 3.04 - 4.16 |
| **Overall 2^nd^ degree relatives** | 1243 | 169546 |  | 3773 | 914083 | 1.78 | 1.63 - 1.94 |
| Grandchildren | 157 | 22969 |  | 602 | 146828 | 1.68 | 1.4 - 2.01 |
| Grandparent | 118 | 24055 |  | 317 | 117908 | 1.82 | 1.46 - 2.27 |
| Nieces/Nephew | 530 | 61920 |  | 1556 | 340941 | 1.86 | 1.67 - 2.08 |
| Aunt/Uncle | 438 | 60602 |  | 1298 | 308406 | 1.73 | 1.53 - 1.97 |
| **Overall 3^rd^ degree relatives** | 2295 | 474160 |  | 9911 | 2569039 | 1.25 | 1.18 - 1.33 |
| Great Grandchildren | 106 | 24834 |  | 533 | 163997 | 1.32 | 1.06 - 1.65 |
| Great Grandparent | 78 | 37015 |  | 323 | 186046 | 1.18 | 0.90 - 1.56 |
| Great Nieces or Nephew | 583 | 115344 |  | 2525 | 651905 | 1.28 | 1.15 - 1.42 |
| Great Aunts or Uncle | 462 | 117069 |  | 1802 | 594602 | 1.28 | 1.14 - 1.44 |
| First Cousin | 1066 | 179898 |  | 4728 | 972489 | 1.23 | 1.12 - 1.34 |
| **Overall 5th degree relatives^b^** | 5729 | 1076761 |  | 28955 | 5780913 | 1.06 | 1.01 - 1.11 |

| **Supplementary Table S5. Sensitivity of familial risk of suicide to removal of families with multiple sibling suicides in first degree relatives.** | | | | | | | | | | |
| --- | --- | --- | --- | --- | --- | --- | --- | --- | --- | --- |
|  |  |  | **Model 1^a,b^** | |  | **Model 2^a,c^** | |  | **Model 3^a,d^** | |
| **Relative-proband relationship** | **Sex of Proband** | **Sex of Relatives** | **Hazard Ratio** | **95% CI** |  | **Hazard Ratio^1^** | **95% CI** |  | **Hazard Ratio^2^** | **95% CI** |
| Sibling-Sibling | Female | Female | 5.80 | 3.34, 10.08 |  | 5.52 | 3.14, 9.71 |  | 4.43 | 2.54, 7.72 |
|  |  | Male | 3.99 | 3.04, 5.24 |  | 3.83 | 2.93, 5.00 |  | 3.51 | 2.69, 4.59 |
|  | Male | Female | 3.94 | 2.98, 5.20 |  | 3.77 | 2.87, 4.96 |  | 3.46 | 2.63, 4.56 |
|  |  | Male | 3.39 | 2.83, 4.05 |  | 3.37 | 2.82, 4.03 |  | 2.87 | 2.41, 3.41 |
| Child-parent | Female | Female | 5.18 | 3.05, 8.77 |  | 5.18 | 3.06, 8.78 |  | 5.15 | 3.04, 8.72 |
|  |  | Male | 4.61 | 3.34, 6.35 |  | 4.61 | 3.34, 6.35 |  | 4.61 | 3.35, 6.35 |
|  | Male | Female | 2.52 | 1.69, 3.74 |  | 2.51 | 1.69, 3.73 |  | 2.45 | 1.64, 3.66 |
|  |  | Male | 3.26 | 2.67, 3.97 |  | 3.26 | 2.68, 3.97 |  | 3.30 | 2.70, 4.02 |
| Parent-Child | Female | Female | 6.33 | 3.55, 11.31 |  | 6.33 | 3.55, 11.32 |  | 6.35 | 3.55, 11.35 |
|  |  | Male | 2.55 | 1.71, 3.81 |  | 2.55 | 1.71, 3.81 |  | 2.55 | 1.71, 3.80 |
|  | Male | Female | 4.77 | 3.32, 6.86 |  | 4.77 | 3.32, 6.86 |  | 4.77 | 3.32, 6.84 |
|  |  | Male | 2.84 | 2.30, 3.50 |  | 2.84 | 2.31, 3.50 |  | 2.80 | 2.28, 3.43 |
| ^a^Model adjusted for relative’s sex and birth year and proband’s sex | | | | | | | | | | |
| ^b^Overall model | | | | | | | | | | |
| ^b^Families with four or more siblings who died by suicide removed from the analysis (N= 4 families) | | | | | | | | | | |
| ^c^Families with three or more siblings who died by suicide removed from the analysis (N = 43 families) | | | | | | | | | | |

Supplementary Figure S1. An example of a matching cluster with two potential suicide probands (d,f, gray colored) in a case family. Squares and circles denote male and female, respectively. Figure is taken with permission from Lee et al. (2013).^26^


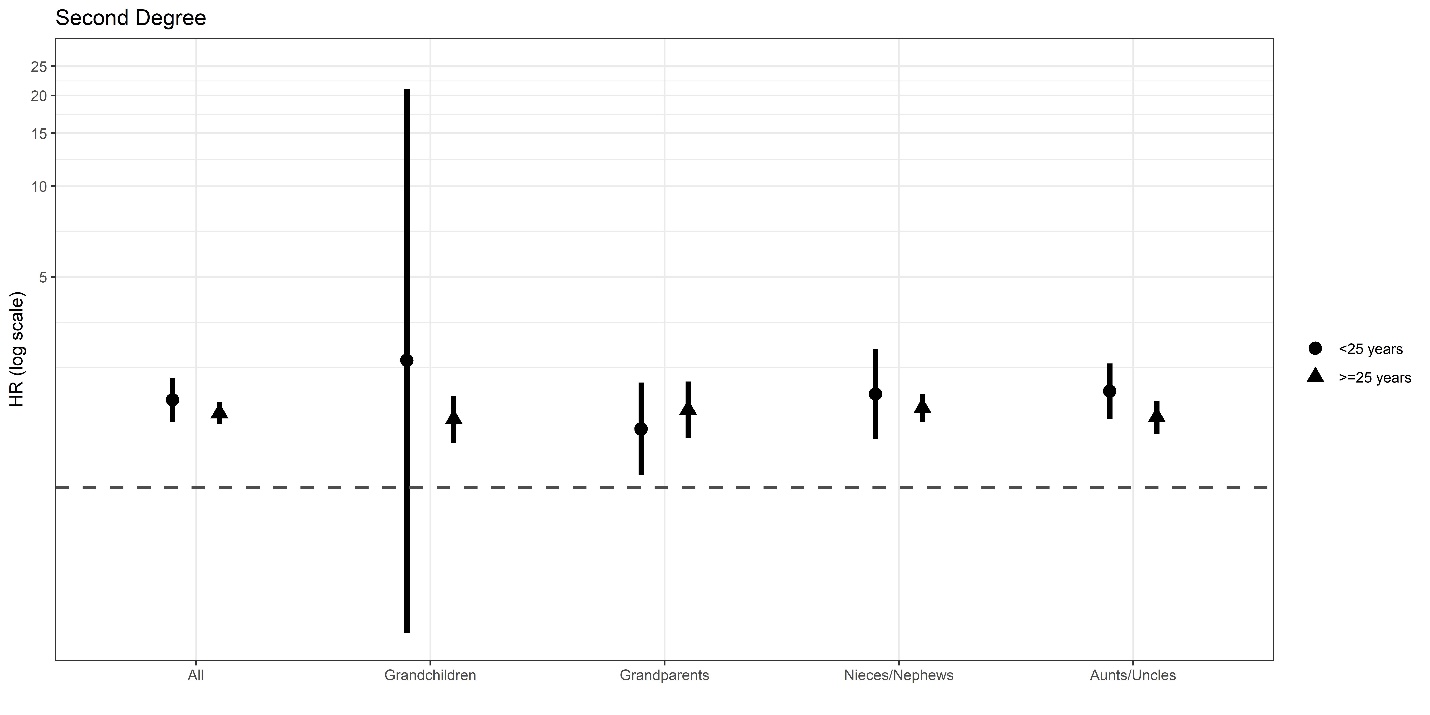


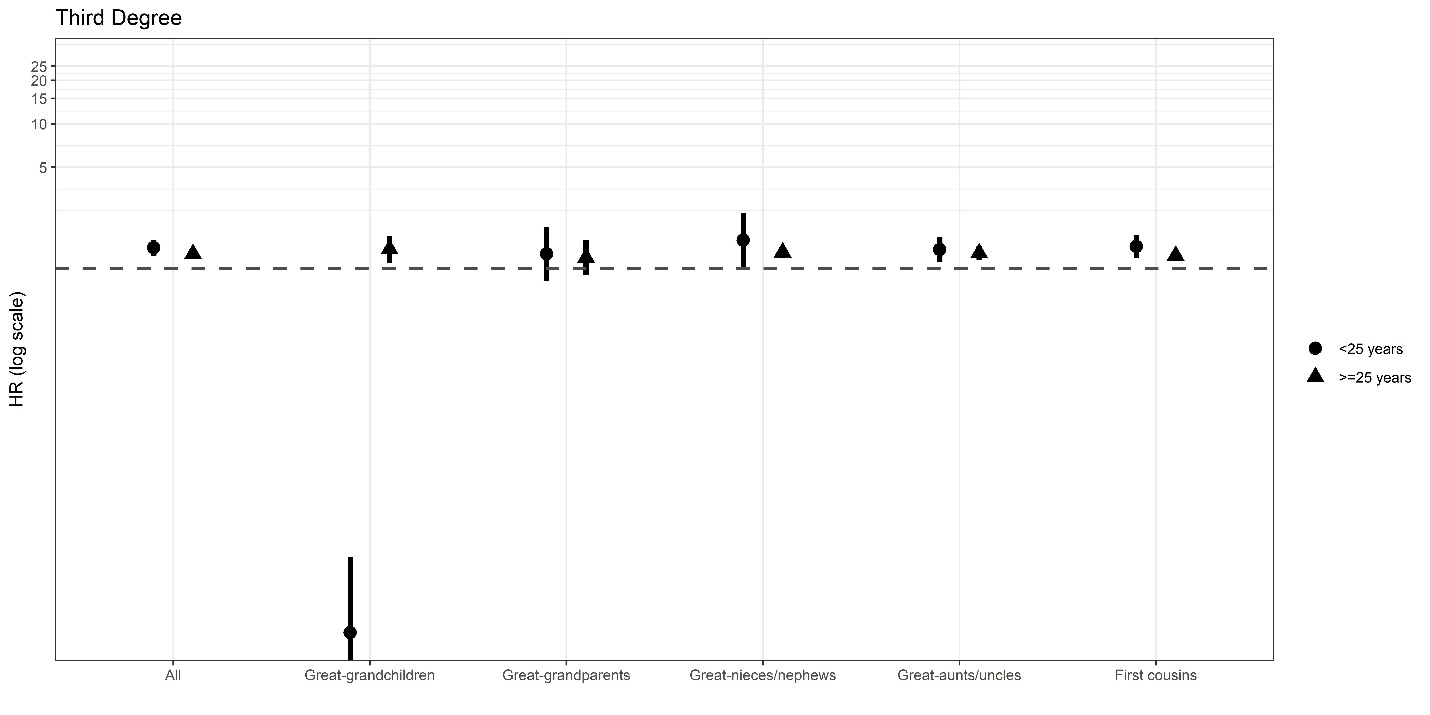


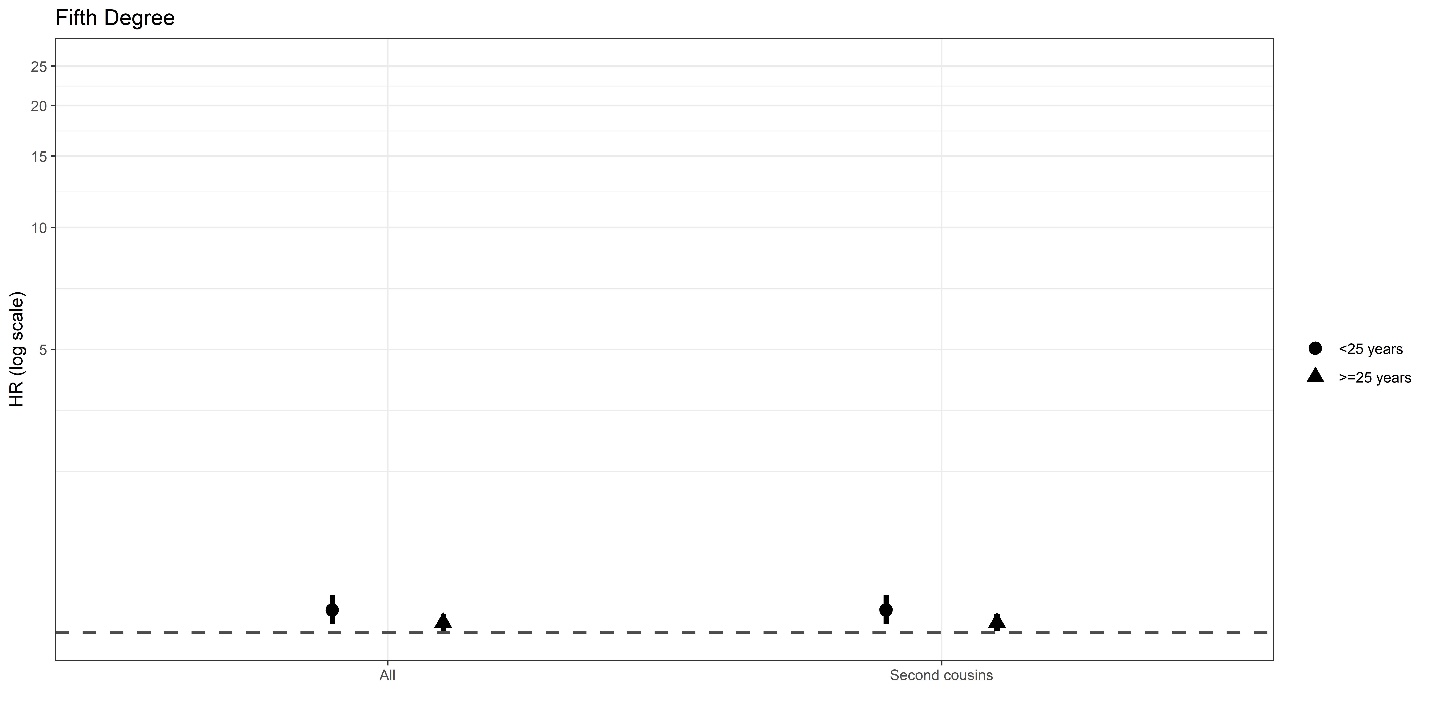


Supplementary Figure S2. Suicide hazard ratios (HR) ±95% confidence intervals (y-axis) in first, second, third, and fifth degree relatives of suicide cases versus controls stratified by <25 years versus ≥25 years of age at time of death. Relative of the proband is on the x-axis. The models were adjusted for relative’s sex and birth year.
